# The differential binding and biological efficacy of auxin herbicides

**DOI:** 10.1002/ps.7294

**Published:** 2022-12-16

**Authors:** Justyna Prusinska, Veselina Uzunova, Paul Schmitzer, Monte Weimer, Jared Bell, Richard M. Napier

**Affiliations:** ^1^ School of Life Sciences University of Warwick Coventry UK; ^2^ Corteva Agriscience Crop Protection Discovery & Development Indianapolis Indiana USA

**Keywords:** auxin, herbicide, *Arabidopsis*, TIR1/AFB, structure activity relationship (SAR)

## Abstract

**Background:**

Auxin herbicides have been used for selective weed control for 75 years and they continue to be amongst the most widely used weed control agents globally. The auxin herbicides fall into five chemical classes, with two herbicides not classified, and in all cases it is anticipated that recognition in the plant starts with binding to the Transport Inhibitor Response 1 (TIR1) family of auxin receptors. There is evidence that some classes of auxins act selectively with certain clades of receptors, although a comprehensive structure–activity relationship has not been available.

**Results:**

Using purified receptor proteins to measure binding efficacy we have conducted quantitative structure activity relationship (qSAR) assays using representative members of the three receptor clades in *Arabidopsis*, TIR1, AFB2 and AFB5. Complementary qSAR data for biological efficacy at the whole‐plant level using root growth inhibition and foliar phytotoxicity assays have also been analyzed for each family of auxin herbicides, including for the *afb5‐1* receptor mutant line.

**Conclusions:**

Comparisons of all these assays highlight differences in receptor selectivity and some systematic differences between results for binding *in vitro* and activity *in vivo*. The results could provide insights into weed spectrum differences between the different classes of auxin herbicides, as well as the potential resistance and cross‐resistance implications for this herbicide class. © 2022 The Authors. *Pest Management Science* published by John Wiley & Sons Ltd on behalf of Society of Chemical Industry.

## Graphical abstract

The natural auxin indoleacetic acid inside its binding pocket in the receptor TIR1. Synthetic auxin herbicides bind with variable affinities which do not all correlate with herbicide efficacies.

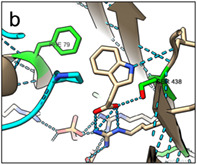

## 1 INTRODUCTION

Auxin herbicides [Auxin Mimics; Herbicide Resistance Action committee (HRAC) group 4, https://hracglobal.com/files/HRAC_MOA_Poster_January_6_2022.pdf] imitate the natural plant hormone indole‐3‐acetic acid (IAA), overloading endogenous auxin response systems to result in plant death. This mechanism of action has been used for weed control for 75 years. Generally, auxin herbicides are used to control broad‐leaved weeds in grass crops, although quinclorac and florpyrauxifen‐benzyl do control some grass and sedge species.^1^ Over this 75‐year period the number of subclasses of auxin herbicides has increased from the original phenoxy‐carboxylate 2,4‐dichlorophenoxyacetic acid (2,4‐D) to include benzoates, pyridine‐carboxylates, pyridyloxy‐carboxylates and quinolone‐carboxylates. The most recent registrations, halauxifen‐methyl and florpyrauxifen‐benzyl, have been classified as pyridine‐carboxylates by HRAC. However, the additional substituted aryl group distinguishes these compounds from other pyridine‐carboxylates and they will be referred to in this paper as 6‐arylpicolinates as in previous publications.^1^, ^2^, ^3^

Auxins are the third most widely used mode of action in global agriculture, behind acetolactate synthase inhibitors and the 5‐enolpyruvylshikimate‐3‐phosphate synthase inhibitor, glyphosate.^1^ Despite being the first auxin herbicide on the market, 2,4‐D remains the most widely used and the use of 2,4‐D along with dicamba may continue to rise because resistances to these auxins have been engineered into crops.^4^, ^5^ Given the importance of auxin herbicides to global food production systems it is important that details of their molecular recognition are understood. Only with this knowledge will we be in a position to manage the threat of target site‐based resistances in weed populations and sustain the control offered by these products.

Auxins are recognized by a small family of nuclear‐localized receptor proteins, Transport Inhibitor Response 1 (TIR1) and the Auxin F‐Box (AFB) proteins.^6^, ^7^ The structure of TIR1 in complex with IAA has been solved by protein crystallography.^8^ After auxin binds to the receptor, a co‐receptor protein binds over the top of the auxin, trapping it in a deep auxin‐binding pocket. These co‐receptors are the Aux/IAA proteins, a family of transcriptional regulators which are ubiquitinated as a consequence of binding and targeted for rapid degradation in the cell's proteasome.^9^ The loss of Aux/IAA proteins allows expression of auxin‐regulated genes to proceed, giving rise to the multiple and diverse responses attributed to auxin action.

The small family of TIR1 and AFB receptors (TIR1 and AFB1‐5) confers functional redundancy.^10^, ^11^ There is also evidence of some specialization in ligand selectivity and differences in dose dependence between members of the TIR/AFB family.^11^, ^12^, ^13^, ^14^ Of particular interest is the selectivity of AFB5 for pyridine‐carboxylate auxin herbicides,^11^, ^15^ whereas indole‐3‐methyltetrazole has been reported to be selective for TIR1.^16^ There has been little investigation into how other auxin scaffolds map onto the receptor clades.

Quantitative structure activity relationship (qSAR) assays using purified TIR1 and AFB5 have been reported,^17^, ^18^ although the collection of compounds used was small compared to the chemical space explored in screens for novel auxins.^19^ The early auxin qSAR work used whole‐plant bioassays and the data were converted into chemical models for the auxin‐binding site.^19^, ^20^, ^21^ These models have stood up well to the test of time, although the activity data measured by whole‐plant bioassays necessarily incorporated contributions from the combined constraints of auxin transport, metabolism and rates of response. Only recently has it been possible to reduce this complexity by using purified receptor proteins. We are now able to contrast and compare structure–activity profiles of the pure receptor with those of whole plants.

Despite formative reports on receptor specificity, the full range of commercial auxins has not previously been tested and no binding data have been reported for the third receptor subclass which is represented in this work by AtAFB2. Further, biochemical binding data have not previously been evaluated against *in vivo* biological efficacy data. If we are to use receptor subclass pharmacology in the development of novel auxins, whole‐plant efficacy data needs to be incorporated into qSAR models for all active compounds. Such comparative data sets may also help us to manage the increasing threat of resistance to auxin herbicides.^1^, ^22^

In this work we have used a representative of each auxin subfamily to assess binding efficacy, with purified receptors representing the three subclasses of the TIR1 family, namely TIR1, AFB2 and AFB5. Also, two whole‐plant bioassays were performed, namely inhibition of primary root extension in seedlings of *Arabidopsis* and phytotoxicity in rosette leaves of *Arabidopsis* after foliar application. Some auxins showed similar activity against all receptors, other auxins bound far more strongly to a particular receptor class and others displayed overall poor binding. Differences in whole‐plant responses were also recorded and we have compared these to receptor binding profiles, providing a holistic view of auxin herbicide efficacies.

## 2 MATERIALS AND METHODS

### 2.1 Chemicals

Technical 2‐methyl‐4‐chlorophenoxyacetic acid (MCPA), mecoprop, dicamba, picloram and aminocyclopyrachlor were purchased from Sigma‐Aldrich (St Louis, MO, USA) and technical fluroxypyr was purchased from Chem Service, Inc. (West Chester, PA, USA). All other technical materials were supplied from Corteva Agriscience, Indianapolis. IN.

### 2.2 Protein expression and purification

The sequences of *Arabidopsis TIR1, AFB2, AFB5* and Arabidopsis SKP1‐like (*ASK1*) genes were codon‐optimized for expression in insect cells, ordered as gBlocks (Integrated DNA Technologies, Leuven, BE) and cloned into pOET5 transfer vector (Oxford Expression Technologies, Oxford, UK) to allow simultaneous expression of each auxin receptor gene (*TIR1*, *AFB2*, *AFB5*) with Arabidopsis SKP1‐like (*ASK1*; Fig. S1) as reported for crystallography and previous *in vitro* binding assays with this complex.^3^, ^8^ Both TIR1/AFB and ASK1 proteins were tagged for purification on His‐Trap columns and TIR1/AFB proteins had an additional FLAG affinity tag. Recombinant baculoviruses were generated in *Spodoptera frugiperda Sf9* cells using pOET5 transfer vectors, Flashback ULTRA DNA and baculoFECTIN II (Oxford Expression Technologies) according to the manufacturer's protocol. Subsequently, the viruses were amplified and quantified using plaque assay and protein expression was optimized in *Trichoplusia ni Hi5* cells. Cells were infected at a density of 1 × 10^6^ cells/mL with multiplicity of infection 2.5, harvested by centrifugation 48 h post infection and stored at −80 °C. To allow for efficient protein purification, AtTIR1, AtAFB2 and AtAFB5 were expressed with an N‐terminal polyhistidine‐enhanced Green Fluorescent Protein‐FLAG‐Tobacco Etch Virus (His10‐eGFP‐FLAG‐TEV) cassette, whereas AtASK1 had only an N‐terminal His10‐(TEV) tag (Fig. S1).

### 2.3 Cell lysis and protein extraction

Cell pellets were lysed for 30 min whilst rolling at 4 °C in a lysis buffer containing Cytobuster (Invitrogen; 10 mL for the cell pellet harvested from each 250 mL of cell culture), 20 mM Tris–HCl pH 7.4, 200 mM NaCl, 1 mM ethylenediaminetetraacetic acidn (EDTA), 50 μM phytic acid, 1 mM tris(2‐carboxyethyl)phosphine (TCEP), DNAse I (Roche) and protease inhibitors (cOmplete Protease Inhibitor Cocktail Tablets, Roche). The lysate was subjected to sonication (three pulses of 15 s), followed by centrifugation at 20000 rpm at 4 °C for 15 min. The supernatant was then filtered through 0.45‐ and 0.2‐μm Whatman GD/X syringe filters.

### 2.4 Protein purification

The filtered lysate was loaded onto a nickel immobilized metal affinity chromatography column (cOmplete His‐Tag Purification Resin, Roche), washed with 10 column volumes of lysis buffer without Cytobuster and eluted directly onto ANTI‐FLAG® M2 affinity gel (Sigma) with His‐elution buffer (20 mM Tris–HCl pH 7.4, 200 mM NaCl, 1 mM EDTA, 50 μM phytic acid, 1 mM TCEP, 250 mM imidazole). The FLAG column was washed with 10 volumes of FLAG buffer (10 mM HEPES pH 7.4, 150 mM NaCl, 3 mM EDTA, 50 μM phytic acid, 1 mM TCEP, 0.05% Tween 20) and protein eluted with 10 mL of 3X FLAG peptide (Sigma) at 100 ug/mL (Fig. S2). Protein was incubated with TEV protease (prepared as a fusion protein with a polyHistidine tag) at 4 °C on a rolling platform overnight before passing through an IMAC column and collecting the nonadsorbed proteins.

The receptor proteins were highly purified after two‐step affinity purification (Fig. S2) and all the fusion tags were removed by the TEV protease before functional assays.

### 2.5 Surface plasmon resonance assays

Auxin binding assays using surface plasmon resonance (SPR) were performed on a Biacore 2000 as described previously.^3^, ^18^, ^23^ Protein was stored on ice and protein concentrations were assayed by A_280_ nm measurement (nanodrop, Thermo Scientific). Briefly, AtAux/IAA7 degron peptide was immobilized on streptavidin‐coated SPR chips, and binding was measured in the presence of IAA or auxin analogue by recruitment of the TIR1/AFB protein from solution as the co‐receptor complex formed on the chip. Kinetic analyses were performed by single cycle kinetics on a Biacore T200 (Cytiva Life Sciences), titrating compounds against a fixed concentration of TIR1 before injection. The degron peptide density on the chips was controlled so that *R*_max_ < 300 Response UNits (RU).

### 2.6 Foliar efficacy bioassays

Bioassays were carried out with the model species *Arabidopsis thaliana*. The wild type (WT) Col‐0 line was compared to an *afb5‐1* mutant line in a Col‐0 background containing a missense mutation of R609K.^11^ *A. thaliana* seeds were sown on Metro‐mix 360 potting mix (Sungro Horticulture) supplemented with vermiculite and stratified for 3 days at 4 °C in the dark. Flats were moved to a growth chamber (as above) and a plastic lid was placed on the flat. On germination, the lid was removed. After 2 weeks in the growth chamber, seedlings were transplanted into 3‐in. pots with Metro‐mix 360 potting mix and moved to a greenhouse. The greenhouse was maintained at a day/night temperature of 23/22 °C and supplemented with light to complete a 16 h photoperiod. Plants were allowed to recover from transplanting for 1 week prior to applications. *A. thaliana* seedlings were sprayed with test compounds at five rates with four replications. Tests included nontreated and solvent check controls. Individual compound doses varied and were based on plate test data or anecdotal potency. Compounds were formulated as technical material and were initially dissolved in 1 part 97:3 (v/v) acetone: dimethylsufoxide (DMSO) followed by 5 parts 1.5% (v/v) Agridex crop oil concentrate in distilled water to give a final concentration of 1.25% (v/v) Agridex. Formulated compounds were sprayed at a volume of 187 L ha^−1^ at a spray height of 43 cm above the plants with an overhead Mandel track sprayer set with 8002 E Tee jet nozzle. Plants were treated at rosette stage just prior to or at initiation of bolting. After spray application, plants were allowed to dry then moved back to the greenhouse. Plants were grown in the greenhouse (same conditions as above) for 2 weeks until harvest. Visual injury/growth reduction observations were made at 7 and 14 days after treatment (DAT) and scored where 0% = no growth reduction and 100% = complete growth reduction or plant death. At 14 DAT the plants were harvested and dried to collect dry weight (DW) biomass.

### 2.7 Root growth inhibition bioassays

*A. thaliana* seed was surface sterilized in 10 mL of 50% bleach and two drops of Tween‐20 for 12 min and rinsed six times with sterile water. Sterile growth media consisted of half‐strength Murashige and Skoog salts supplemented with 0.4% agar and 0.8% sucrose. In a laminar flow hood, 8 mL of liquid media was transferred to sterile test‐tubes placed in a heat block to maintain media in a liquid state. Compounds were previously diluted in 50/50 DMSO/water to a known molar concentration. A 50‐μL aliquot of diluted compound was added to each tube to give a specified final concentration, normally 10, 1, 0.1, 0.01 or 0.001 μM. A single tube was treated with solvent only as a control. Test compounds were mixed into the media by pipetting up and down three times, then 6 mL of media with test compound was added to one well in a six‐well plate, working from low‐to‐high concentration, and allowed to solidify in the laminar flow hood. Approximately 15 sterilized seeds were placed on the solidified surface and the remaining 2 mL of liquid media was added to cover and disperse seeds. Plates were sealed with paraffin and moved to a Conviron growth chamber set to 60% humidity, 25 °C and light intensity 100 μE m^2^ s^−1^ with a 16 h photoperiod. After 8 days primary root measurements of five random plants were taken by removing plants from media and measuring with a ruler. Treatments were compared against the solvent control to determine percentage root growth inhibition. Each experiment was run twice and averages were combined for analysis.

### 2.8 Data analysis

The data collected in the foliar application of compounds generated a measure of sensitivity or dose response. The data was analyzed by regression in JMP 10.0 and fit to a nonlinear exponential three parameter fit model:

*y = a + b × Exp*(*c × x*).

where *a* is the asymptote, *b* is the scale and *c* is the rate of growth. The model was used to determine the herbicidal dose that caused 50% reduction in growth (GR50). GR50 values were compared between mutant and WT to give an indication of fold resistance over WT (Table 3). A log‐logistic model^24^ was also used to fit the data and gave similar GR50 values with greater standard errors without changing the pattern of responsiveness. Hence, the outputs from the nonlinear exponential three‐parameter fit model are used as the results given in Table 3.

Where applicable, analysis of variance (ANOVA) was applied followed by multiple pair‐wise comparison tests, Tukey–Kramer **honestly significant difference** (HSD), to determine if there were differences between mutants and WT plants.

#### 2.8.1 In silico modelling, chemical and protein visualization

*In silico* modelling, molecular graphics and analyses were performed with the open‐source UCSF Chimera package developed by the Resource for Biocomputing, Visualization, and Informatics at the University of California, San Francisco (supported by NIGMS P41‐GM103311).^25^ Crystal structures 2P1P and 2P1Q^8^ were sourced from the Royal Crystallographic Society B database.^26^ Marvin and ChemDraw Professional v15.0.0.106 was used for drawing, displaying and characterizing chemical structures, substructures and reactions.

Accession numbers for proteins studied were: AtTIR1 (Q570C0), AtAFB1 (Q9ZR12), AtAFB2 (Q9LW29), AtAFB3 (Q9LPW7), AtAFB4 (Q8RWQ8), AtAFB5 (Q9LTX2).

## 3 RESULTS

### 3.1 Active auxin receptors expressed from recombinant baculovirus

The auxin signaling pathway has undergone substantial functional diversification within vascular plants since they diverged from bryophytes.^27^, ^28^ The *Arabidopsis* genome encodes six TIR1/AFB family members grouped in three clades of two paralogs each, a result of multiple gene duplication events.^10^, ^27^, ^28^ Sequence conservation (identical amino acids) between the two most diverse clades is around 50% for the full‐length proteins, 50.4% for AtTIR1 and AtAFB5 (Table 1), rising to 61% identity between AtTIR1 and AtAFB2. However, identity is much greater for the residues lining the binding pocket, 91% and 80% for AFB2 and AFB5 against AtTIR1, respectively. The divergence between pairs in each clade is low, especially in the binding pocket (Table 1). Representatives of each clade, AtTIR1, AtAFB2 and AtAFB5 were cloned into recombinant baculovirus lines for expression and purification before evaluation by binding analysis.

**Table 1:** Sequence identities at the amino acid level for TIR1 homologues in *Arabidopsis thaliana.* Identities are given as percentages. The heat scale runs from green (greatest identity) to red (low identity) with the cells for self *vs* self in grey. (a) Full‐length proteins and (b) binding pocket residues only. The sequences lining the binding pocket were identified from the crystal structure of TIR1 (Supplementary Table S1).^8^

(a)	TIR1	AFB1	AFB2	AFB3	AFB4	AFB5
TIR1		70.3	61.0	60.0	49.0	50.4
AFB1			55.7	55.2	48.1	50.5
AFB2				86.1	50.4	51.4
AFB3					50.2	51.4
AFB4						78.7
AFB5						

(b)	TIR1	AFB1	AFB2	AFB3	AFB4	AFB5
TIR1		91	91.0	89.0	82.0	80
AFB1			84	84	80	77
AFB2				100	82	82
AFB3					82	82
AFB4						100
AFB5						

### 3.2 Binding of auxins to AtTIR1/AFB‐ASK1 complexes

The auxin‐binding activity of purified TIR1/AFB‐ASK1 complexes was tested in SPR experiments^3^ (Fig. 2). All three receptors (*TIR1, AFB2, AFB5*) bound IAA, although the rates of complex association and dissociation varied. Rapid dissociation of the IAA complex has been reported before for AFB5,^17^, ^18^ but the kinetics of binding are also distinct for AFB2. Dissociation of the receptor complex was slow for AtTIR1, more rapid with AtAFB2 and more rapid again with AtAFB5 (Fig. 2). The widely used auxin 2,4‐D binds to all three receptors, and in all cases binding was weaker than for IAA (29%, 22% and 40% of IAA binding for AtTIR1, AtAFB2 and AtAFB5, respectively). This lower affinity is probably because of more rapid dissociation kinetics with 2,4‐D (after the association phase ends, the binding curve reaches the baseline sooner with 2,4‐D than with IAA), whereas binding and dissociation rates of 1‐NAA are closer to those for IAA (Fig. 2).

An assessment of binding to the three representative receptor families was completed for a wider variety of commercial auxin herbicides (Fig. 3). The benzoate auxin dicamba showed low binding to all three auxin receptors. Three phenoxy‐carboxylate auxins, MCPA, mecoprop and dichlorprop, also showed lower binding to all three receptors compared to IAA, but mecoprop and dichlorprop showed significantly higher binding to TIR1 than 2,4‐D and MCPA. Mecoprop also showed higher binding to AtAFB2 than the other three phenoxy‐carboxylate auxins.

The three pyridine‐carboxylate herbicides, clopyralid, picloram and aminopyralid, showed relatively low binding to all three classes of auxin receptor, but the binding of picloram and aminopyralid was significantly higher to AtAFB5 than to AtTIR1 and AtAFB2. The binding pattern of the pyrimidine‐carboxylate herbicide aminocyclopyrachlor was similar to that of picloram and aminopyralid. The strong selectivity of picolinate auxins for AtAFB5 *in vivo* ^11^, ^15^ was manifest in the SPR assay as a very strong, fast binding response, for example with the new 6‐arylpicolinate herbicide halauxifen (Figs 2 and S3), resulting in a much stronger response with AtAFB5 than IAA (170% of IAA binding), whereas halauxifen showed little binding with AtTIR1 (10%) and AtAFB2 (<5%). The binding characteristics for florpyrauxifen are similar to those for halauxifen (Fig. S3 and kinetic data below). The experimental 6‐arylpicolinate DAS534 also showed a strong bias for binding to AtAFB5 corresponding well with previously published data.^18^ The pyridyloxy‐carboxylate herbicides triclopyr and fluroxypyr exhibited similar levels of binding for all three clades of auxin receptor, albeit lower binding than IAA. No binding of quinclorac was observed for any of the receptors investigated (Fig. 2; values all below 5%), correcting the result presented previously for this compound on AtAFB5.^18^

To explore the binding responses further, kinetic data were collected for some representative auxins (Table 2). The kinetics of binding are distinctive for each clade of receptors. The kinetic values for AFB2 follow essentially the same pattern as those for TIR1 but with faster dissociation rates (Table S2), leading to slightly poorer affinities.

**Table 2:** Binding kinetics of indole‐3‐acetic acid (IAA) and representative auxin herbicides to *Arabidopsis* TIR1, AFB5 and AFB2. Averaged values for the equilibrium dissociation constant (Ave KD) are given.

Treatment	Ave KD (μM)		
	AtTIR1	AtAFB2	AtAFB5
IAA	7.9	12.6	33
2,4‐D	229	392	152
Florpyrauxifen	62	105	2.7
Halauxifen	128	333	2.4
Fluroxypyr	18	45	95
Picloram	319	ND	107

Abbreviation: Average values are shown (data statistics in Table S2). 2,4‐D, 2,4‐dichlorophenoxyacetic acid; ND, not determined with statistical confidence.

The natural auxin IAA had a relatively high affinity (low KD) for all three receptors, although the affinity of IAA was 4‐fold higher for TIR1. Given the high auxin activity of 2,4‐D in many biological assays (see Fig. 5), the phenoxy‐carboxylate 2,4‐D had surprisingly low affinity for all three receptors due primarily to its much faster dissociation rate constants (Table S2). The 6‐arylpicolinates, halauxifen and florpyrauxifen, showed high affinities for AFB5 which were conferred by both faster association and slower dissociation rates. Thus, the affinity of these herbicides for AFB5 is greater than the affinity of the natural auxin IAA for TIR1. Fluroxypyr is an interesting intermediate, with moderate affinities for the three receptors and comparatively slow dissociation rate constants. Picloram showed low binding to all three receptors, although binding to AFB5 was higher than to TIR1 and AFB2. Overall, the kinetic data emphasized again the much stronger binding of the picolinate class with AFB5, even for the smaller molecules in the family like picloram. It was of interest that this preference broke down when the carboxylic acid group was presented as the oxyacetate group, as in triclopyr and fluroxypyr. In these cases, binding to TIR1 and AFB2 was elevated to levels similar to that for AFB5 rather than binding being lost by AFB5.

### 3.3 Phytotoxicity of auxin subclasses on *A. thaliana*

Having established the pharmacology of the three receptor clades in *A. thaliana*, a set of compounds was also evaluated for foliar phytotoxicity on *A. thaliana* plants (Fig. 4 and Table 3) and for inhibition of primary root growth of *A. thaliana* seedlings (Fig. 5). Comparisons of growth inhibition after foliar application of auxins to WT and *afb5‐1* ^11^ mutant seedlings further clarified the specificity of auxin chemistries and clear visual resistance to specific auxin compounds was observed (Fig. 4 and Table 3). Results from foliar applications of picloram and 2,4‐D were similar to previous reports with resistance observed in *afb5‐1* to the pyridine‐carboxylate, but not to 2,4‐D.^11^ Resistance was not complete, and auxinic effects such as epinasty, stunting and lack of apical dominance were still observed in the mutant (Fig. 4). Effects were dose dependent, allowing for calculation of GR50 values by regression analysis and an overall fold resistance over WT (resistance ratio) from both DW and visual phytotoxicity data (Table 3).

**Table 3:** Growth reduction comparisons of foliar applied auxin herbicides on *Arabidopsis* wild‐type (WT) and the *afb5‐1* mutant line

Compound	Class	Type	GR50 (DW % of NT) g ai ha^−1^	SE	Resistance ratio (afb5‐1 *vs* WT)	GR50 (visual injury) g ai ha^−1^	SE2	Resistance ratio (afb5‐1 *vs* WT)^3^
2,4‐D	Phenoxy‐carboxylate	WT	**3.9**	1.6		**19.2**	1.5	
		*afb*5‐1	**9.9**	3.6	**2.5**	**40.5**	4.1	**2.1**
Dicamba	Benzoate	WT	**16.5**	5.3		**169**	13.6	
		*afb*5‐1	**60.8**	18.1	**3.7**	**290**	20.5	**1.7**
Clopyralid	Pyridine‐carboxylate	WT	**497**	187.1		**2698**	162	
		*afb*5‐1	**3999**	2.6	**8**	**4259**	ND	**1.6**
Triclopyr	Pyridoxy‐carboxylate	WT	**4.7**	0.9		**31.8**	3.8	
		*afb*5‐1	**18.4**	4.6	**3.9**	**41.8**	4.9	**1.3**
Aminopyralid	Pyridine‐carboxylate	WT	**3.4**	1.2		**40.1**	2.7	
		*afb*5‐1	**68.7**	30.6	**20.2**	**ND**	ND	**ND**
Picloram	Pyridine‐carboxylate	WT	**13.7**	4.2		**202**	14.1	
		*afb*5‐1	**996**	ND	**72.7**	**ND**	ND	**ND**
Fluroxypyr	Pyridoxyl‐carboxylate	WT	**35.7**	12.8		**234**	13.3	
		*afb*5‐1	**84.1**	26.4	**2.4**	**310**	13.5	**1.3**
Aminocyclopyrachlor	Pyridine‐carboxylate	WT	**4.4**	1.9		**7**	1.4	
		*afb*5‐1	**8.3**	4	**1.9**	**36.8**	10.1	**5.3**
DAS5534	6‐Aryl picolinates	WT	**ND**	ND		**0.4**	0.04	
		*afb*5‐1	**1.2**	0.2	**ND**	**4.3**	0.4	**10.8**
Halauxifen	6‐Aryl picoliante	WT	**0.08**	0.01		**0.4**	0.03	
		*afb*5‐1	**0.6**	0.1	**7.5**	**2.4**	0.2	**6**
Florpyrauxifen	6‐Aryl picolinate	WT	**0.1**	0.02		**0.7**	0.04	
		*afb*5‐1	**1**	0.3	**10**	**5.2**	0.4	**7.4**
Quinclorac	Quinoline‐carboxylate	WT	**1015**	ND		**ND**	ND	
		*afb*5‐1	**1002**	2	**1**	**ND**	ND	**ND**

The bold values are the key data column and the bold helps the reader pick out the important data from the background details.

Abbreviation: 2,4‐D, 2,4‐dichlorophenoxyacetic acid; DW, dry weight; GR50, rate of herbicide that provides 50% reduction in DW or 50% visual injury; ND, a value could not be determined with statistical confidence based on the rates tested; NT, not treated; WT, wild type.

Of the pyridine‐carboxylates, the *afb5‐1* mutant was most tolerant to picloram followed by aminopyralid and clopyralid with respective DW resistance ratios of 72.7, 20.2 and 8.0. Interestingly, WT *A. thaliana* has inherent tolerance to clopyralid, which may explain why clopyralid can be utilized for weed control in other Brassica species. The *afb5‐1* mutant also exhibited resistance to the 6‐arylpicolinate herbicides florpyrauxifen, halauxifen and DAS534 (DW resistance ratios of 10, 7.5 and not determined, respectively). A DW resistance ratio could not be calculated for DAS534 as a WT GR50 could not be extrapolated from the treatment doses given, but visual grades indicated a resistance ratio of 10.8.

Resistance of *afb5‐1* mutants to the pyridine carboxylates confirmed their tendency for binding to the AFB5 receptor, as previously noted.^11^ Low binding of these herbicides to other TIR/AFB receptors is likely to be the cause of the remaining dose‐dependent plant responses in the *afb5‐1* mutants. The *afb5‐1* mutant sensitivity was closer to WT when phenoxy and pyridyloxy compounds 2,4‐D, fluroxypyr and triclopyr were applied (resistance ratios of 2.5, 2.4 and 3.9, respectively). Fluroxypyr bound to all three receptors approximately an order of magnitude more strongly than 2,4‐D (Table 2), yet the GR50 value for 2,4‐D was considerably lower than that of fluroxypyr. The benzoic acid compound dicamba exhibited a similar resistance pattern at 3.7‐fold resistance. The lack of resistance to these compounds suggests that they do not primarily act *via* AFB5, but rather interact with other auxin receptors equally well or have specific preference for other auxin receptors (TIR1 and AFB2) in the case of 2,4‐D.^29^

Overall, quinclorac did not provide much phytotoxicity to the WT or *afb‐5* mutant plants, signifying an intrinsic resistance mechanism in *A. thaliana*. Commercial quinclorac use is directed mainly at monocot weed control, with some dicot susceptibility. The lack of herbicidal effects on *A. thaliana* is not necessarily surprising as quinclorac is utilized for weed control in *Brassica* crops.

Not many dose studies on auxins have used whole‐rosette phytotoxicity assays and so for comparison we added measurements of the inhibition of seedling primary root growth, a standard auxin activity assay (Fig. 5). The dose–response results using WT and *afb5‐1* lines followed similar trends to those from foliar applications. In general, the 6‐arylpicolinates caused strong root growth inhibition, therefore the dosage was adjusted to bring the response curves into the range of the other compounds tested (Fig. 5b). No resistance was detected for dicamba, 1‐NAA and 2,4‐D (Fig. 5a), nor to the pyridyloxy‐carboxylates or to the quinolate, quinclorac (Fig. 5c), whereas the *afb5‐1* mutant exhibited considerable resistance to halauxifen and florpyrauxifen (Fig. 5b), and the pyridine and pyrimidine carboxylic acid molecules picloram, clopyralid, aminopyralid and aminocyclopyrachlor at higher doses (Fig. 5d). The low level of phytotoxicity of picloram did not conform to previous reported results,^11^ but this assay was repeated multiple times with different compound lots with no change in outcome. Quinclorac caused very low root growth inhibition and only at the highest rate tested (10 μM).

### 3.4 Auxin‐binding pocket sequences and pharmacological grouping

**Figure 1:**
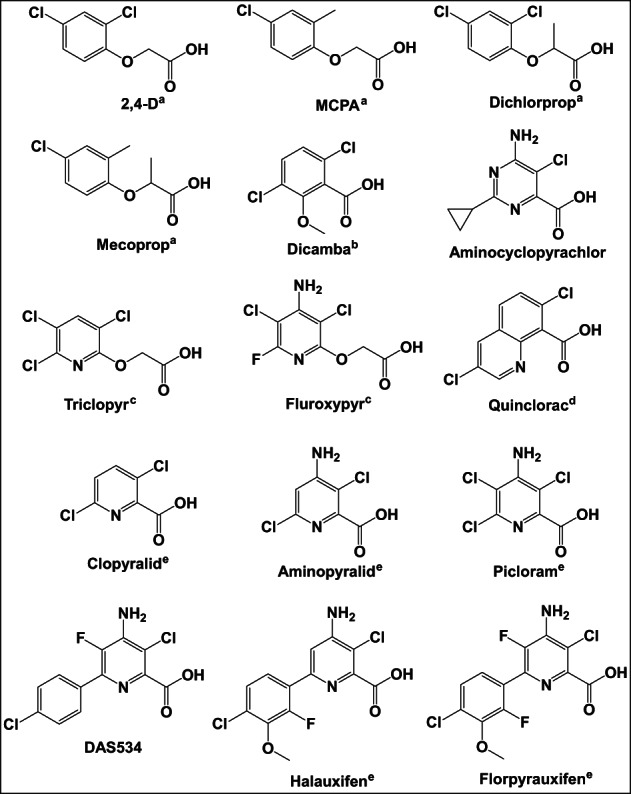
Auxin herbicide molecules from all known chemical classes that were utilized in the study: (a) phenoxy‐carboxylates, (b) benzoate, (c) pyridyloxy‐carboxylates, (d) quinoline‐carboxylateand and (e) pyridine‐carboxylates. Aminocyclopyrichlor and DAS534 are not classified by HRAC. 2,4‐D, 2,4‐dichlorophenoxyacetic acid; DAS534, MCPA, 2‐methyl‐4‐chlorophenoxyacetic acid.

It is instructive to compare the pharmacology with receptor sequences and the structure of AtTIR1 provided by crystallography (Fig. 6).^8^ A number of binding pocket residues differ between the TIR1/AFB1 clade and the AFB4/5 clade, and these could contribute to the distinct auxin binding specificity profiles (Table S1). When these residues are mapped onto the structure, most of their side chains are found to face away from the pocket and probably contribute little to ligand selections. However, Phe79 and Ser438 both have side chains within 5 Å of IAA when bound, and their substitutions in AtAFB5 are likely to make more substantial contributions to the changed selectivity of this clade for auxins. The change of Phe79 into arginine changes an aromatic ring system for a polar aliphatic residue, resulting not only in a change in charge at that side of the binding pocket, but importantly also results in more space in the binding site (Fig. 6a,c,d), space which is likely to be conducive to the binding of the larger 6‐arylpicolinate auxins (Fig. 1). However, given that the canonical picolinate, picloram, is composed of only a single aromatic ring and does not extend into the additional space, this space cannot be the principal determinant of the distinct pharmacology of AtAFB5. This space in AFB5 offers great potential for the rational design of auxins.

**Figure 2:**
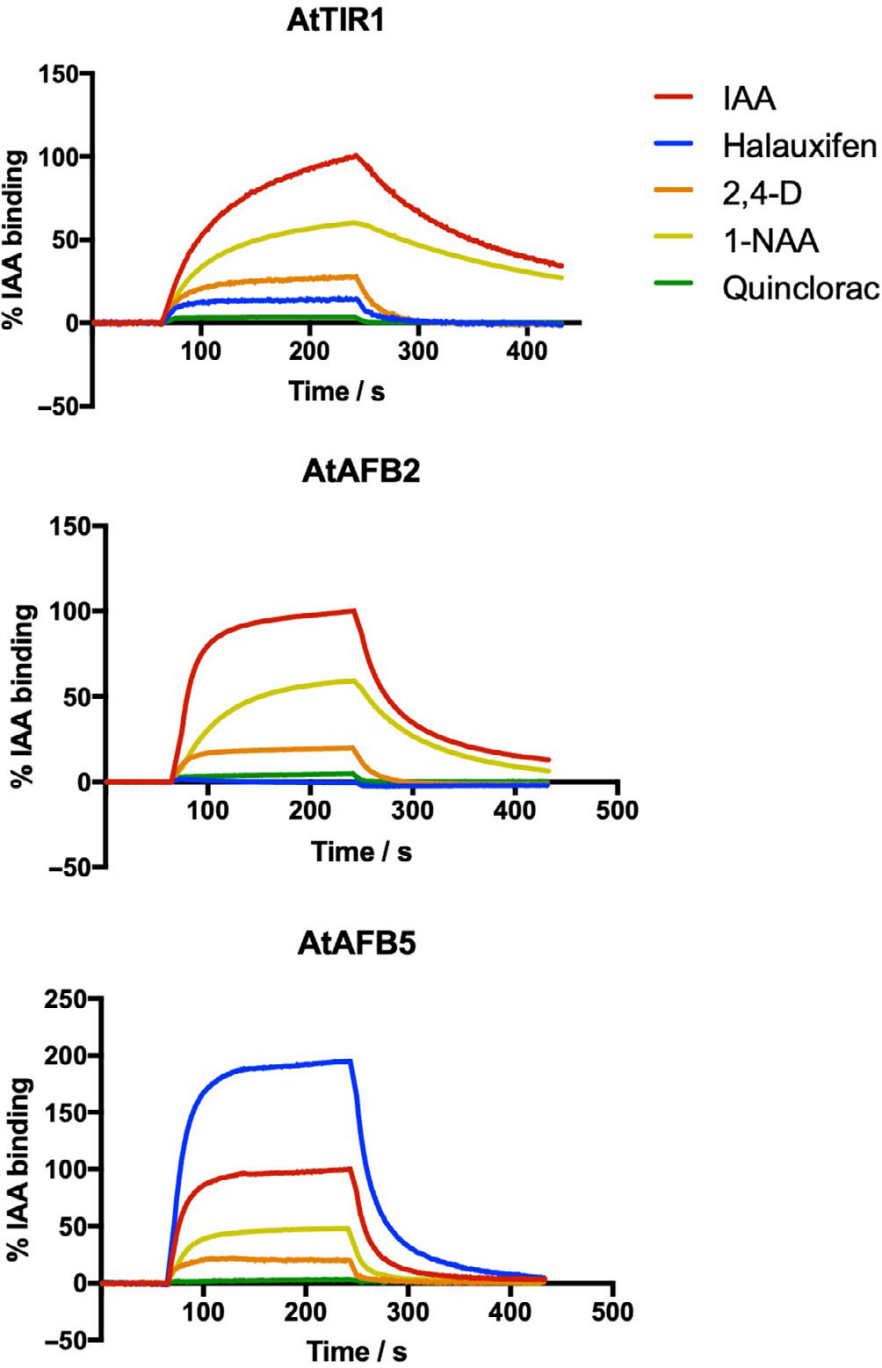
SPR sensorgrams showing binding of AtTIR1, AtAFB2 and AFB5 to the *Arabidopsis* Aux/IAA7 degron peptide in the presence of 50 μM IAA, halauxifen, 2,4‐D, 1‐NAA and quinclorac. The sensorgrams were normalized to the binding of IAA (100%, red) which was included in every screen. 1‐NAA, 1‐napthaleneacetic acid; 2,4‐D, 2,4‐dichlorophenoxyacetic acid; IAA, indole‐3‐acetic acid

The change Ser438 for alanine in AFB5 replaces the polar side group of serine for an uncharged methyl group (Fig. 6b,e,f). In this case, the loss of the hydroxyl loses the contribution of this residue to a hydrogen bond formed with the carboxylic acid group of bound auxins (Fig. 4b). This loss might account for the faster dissociation rates of all auxins from AtAFB5 (Fig. 2).

## 4 DISCUSSION

**Figure 3:**
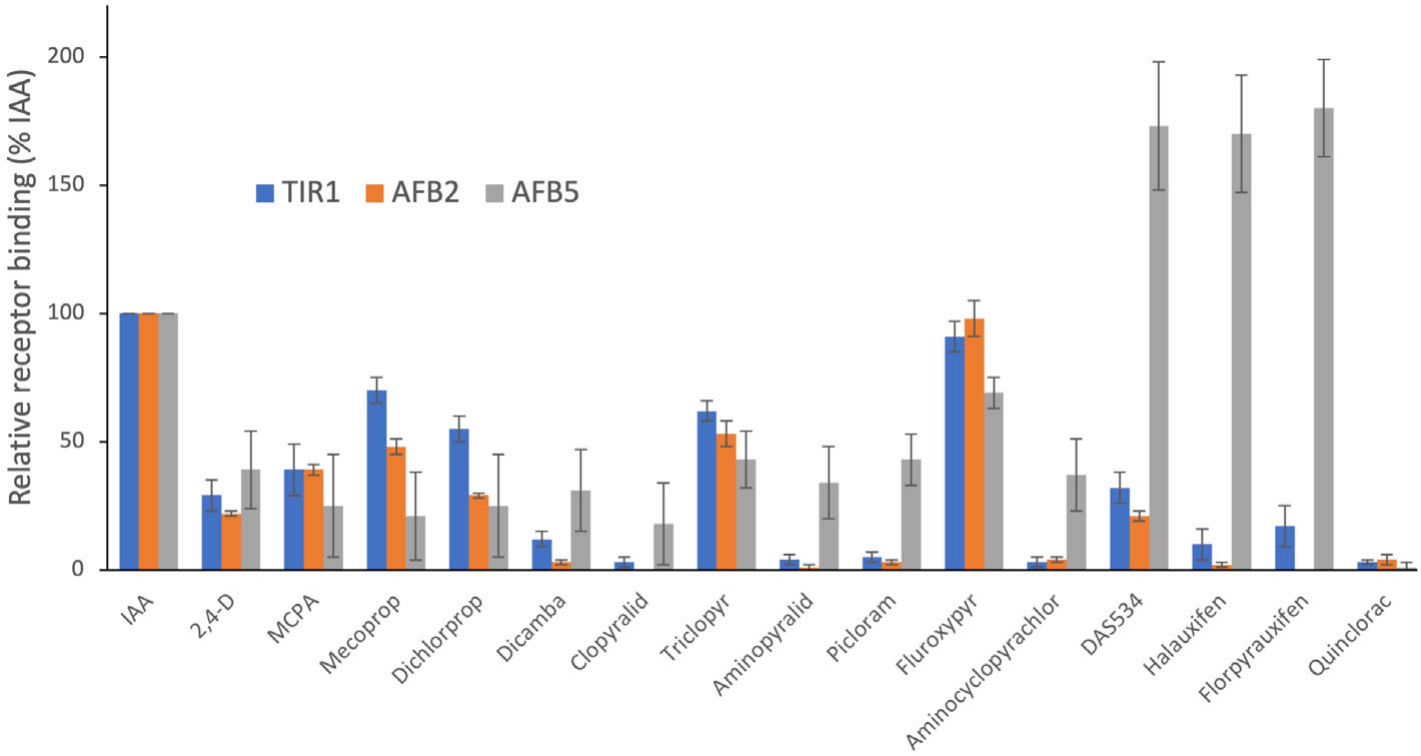
Relative binding of auxin herbicides. Binding was assayed by SPR and in all cases binding amplitudes at the end of the association period were compared to the amplitude for the natural auxin IAA (indole‐3‐acetic acid). All compounds were tested at 50 μM. Average data (*n* minimum 4) are plotted ± SD. 2,4‐D, 2,4‐dichlorophenoxyacetic acid; IAA, indole‐3‐acetic acid; MCPA, 2‐methyl‐4‐chlorophenoxyacetic acid

Structurally, all members of the auxin herbicide class contain a halogenated aromatic ring connected in some manner to a carboxylic acid. These chemical similarities allow us to speculate that they share a target site. The first auxins were synthesized in the early‐1940s, but it was not until 2005 that the molecular target of auxins was elucidated.^10^, ^11^ The identification of TIR1 and AFB receptors as the molecular target of auxins has allowed research to expand into the similarities and differences of auxins *in vitro* and enabled agriculture to use many of the techniques developed by modern pharmacology. A survey of 63 compounds, including multiple auxins, revealed differential binding to AtTIR1 and AtAFB5 for many of the tested compounds.^18^ The current research has focused specifically on the current commercial auxin herbicides, as well as on binding data for AtAFB2. This representative of clade 2 was shown to have binding selectivity very similar to that for TIR1 (Figs 2 and 3).

**Figure 4:**
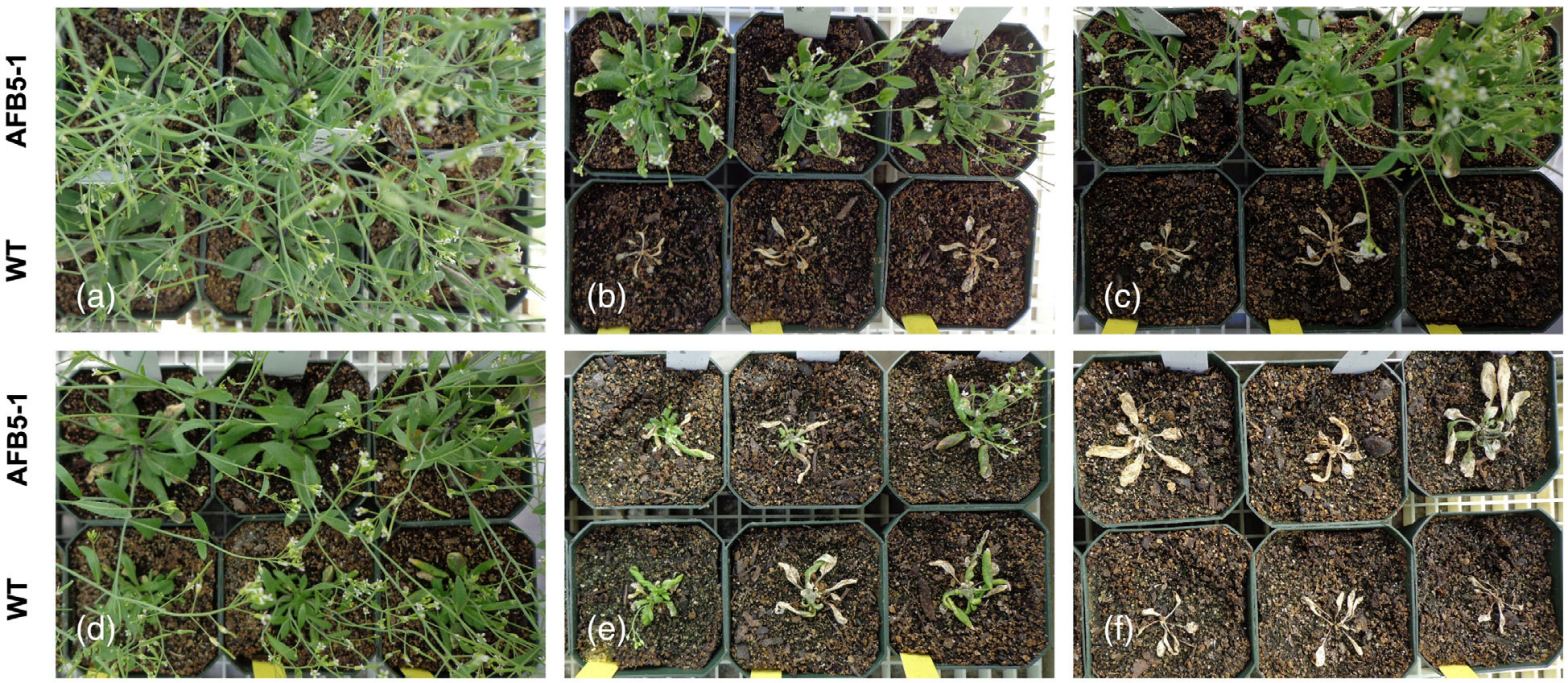
Phytotoxicity symptoms on Arabidopsis thanliana (ARBTH) 14 days after foliar applications of auxins. In each panel, the mutant line afb5‐1 is shown in the top line, wild‐type (WT) Col‐0 in the lower line and three replicates are shown for each. Pictured examples selected based on application rates for which there was a clear difference in efficacy or lack thereof (fluroxypyr, 2,4‐D). (a) non‐treated, (b) 4 g ai ha^−1^ halauxifen, (c) 4 g ai ha^−1^ florpyrauxifen, (d) 200 g ai ha^−1^ picloram, (e) 400 g ai ha^−1^ fluroxypyr and (f) 100 g ai ha^−1^ 2,4‐D. 2,4‐D, 2,4‐dichlorophenoxyacetic acid.

**Figure 5:**
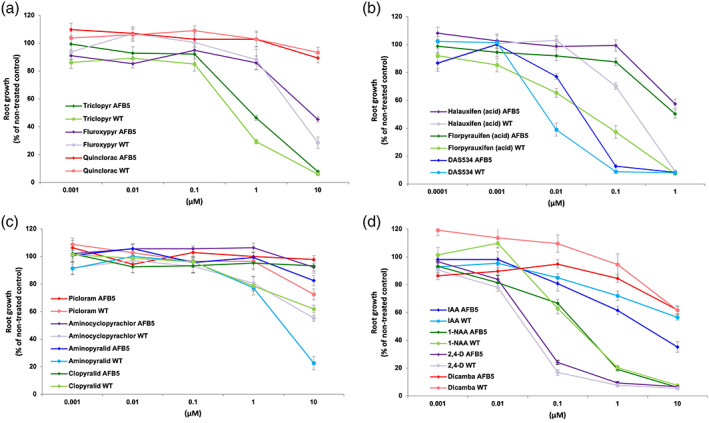
Root growth inhibition by auxin subclasses for both Col‐0 and the mutant line *afb5‐1*. Note that the dose–response curves for the two genotypes are similar for all auxins except for the pyridine carboxylates (b and d). The resistance of *afb5‐1* to the 6‐arylpicolinates is indicated by a shift of the response curve to higher concentrations relative to wild type. Average values ± standard errors are shown. 1‐NAA, 1‐napthaleneacetic acid; 2,4‐D, 2,4‐dichlorophenoxyacetic acid; IAA, indole‐3‐acetic acid; WT, wild type

**Figure 6:**
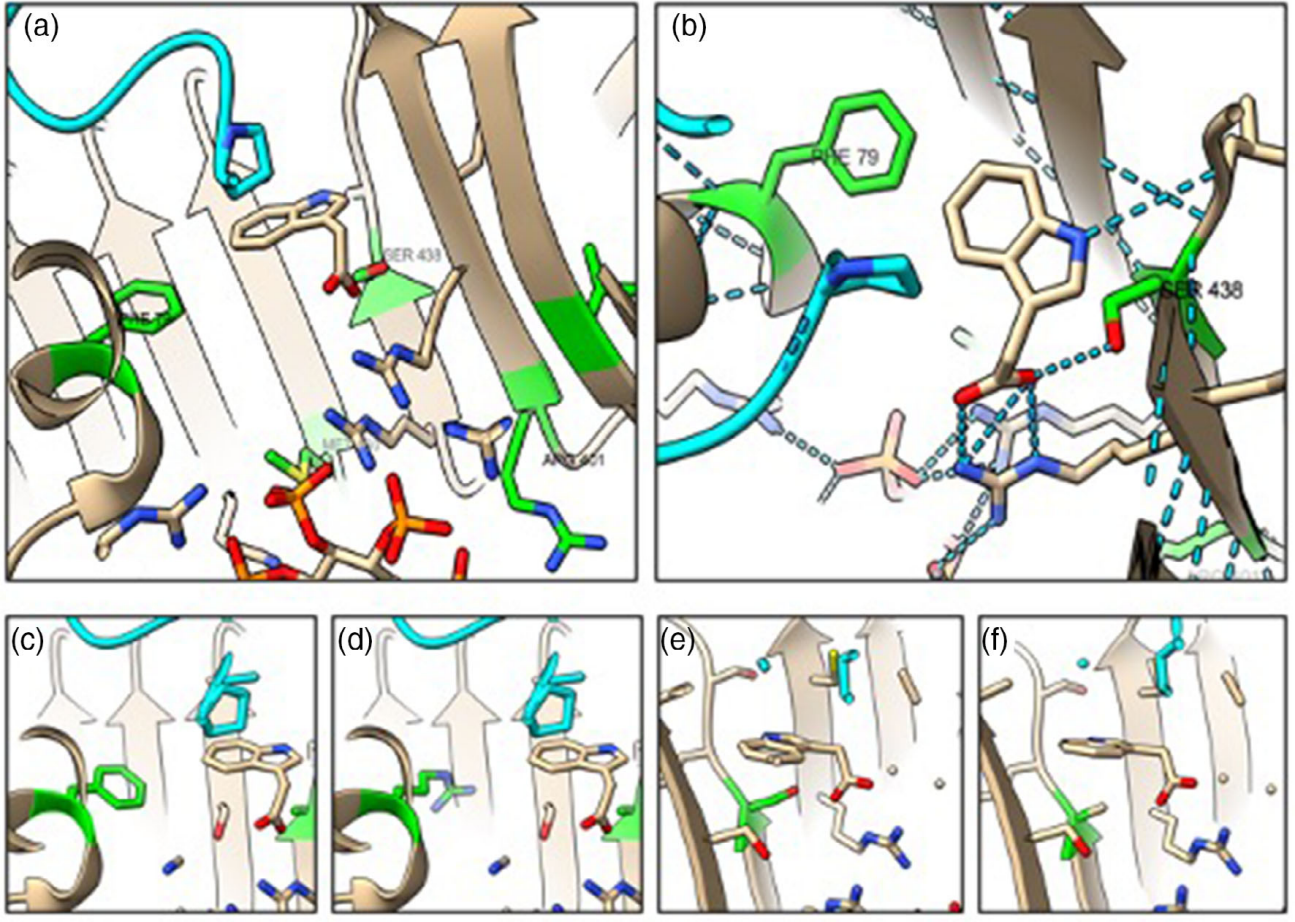
The ligand pose of IAA in AtTIR1 according to the crystal structure (2P1Q). The co‐receptor IAA7 (cyan) traps the auxin in the binding pocket. (a) and (b) Key residues in the binding pocket of TIR1 colored green if they are not conserved in AtAFB5 (Table S1). Phe79 completes a hydrophobic wall adjacent to the indole ring of IAA. Ser438 is at the opposite wall of the pocket, contributing a hydrogen bond (blue dashed lines) to help position the carboxylic acid of IAA. Met460 and Arg401 are not in close contact with bound IAA. (c–f) The consequences of mutating the AtTIR1 residues to the AtAFB5 sequences. Phenylalanine 79 is changed to arginine (c and d), serine 438 to alanine (e and f).

The results are based on assays using Arabidopsis proteins, but it is reasonable to speculate that the data reflect clade specificities across angiosperms, or at least eudicots. The division of the TIR1/AFBs into three clades has been mapped onto the ancestry of the plant kingdom.^28^ The auxin receptors split from the jasmonate receptor at the origin of land plants. At the origin of the ferns, the AFB4/5 clade split from the TIR1/AFB1‐3 branch and TIR1/AFB1 split from the AFB2/3 clade at the origin of the angiosperms. One other clade, AFB6, also arose at the time of the ferns, but has been lost in many genera, including Arabidopsis. Hence, our data may be considered representative of the three receptor clades that are conserved and necessary throughout the angiosperms.

Analysis of relative binding data (Fig. 3) allows comparisons of large numbers of compounds and identification of binding trends. Kinetic binding assays afford a higher level of pharmacological detail (Table 2) and fewer representative auxins were used in the kinetic study. The low binding of dicamba to all the receptors made acquiring kinetic data for a benzoate problematic. The 6‐arylpicolinates halauxifen and florpyrauxifen showed high affinities (low KD values) for AFB5, with much poorer affinities for TIR1 and AFB2. In contrast, AtTIR1 and AtAFB2 showed their highest affinity toward IAA. Interestingly, only fluroxypyr gave KD values of a similar magnitude and pattern as IAA for all three receptors (Table 2) and the affinity of all three receptors was quite low for both 2,4‐D and picloram. Overall, the 6‐arylpicolinates help distinguish the AtAFB5 clade, whereas the pharmacologies of AtTIR1 and AtAFB2 are similar, albeit with AtAFB2 displaying somewhat poorer affinities due primarily to faster dissociation kinetics (Fig. 2). It is clear that there are many contributory facets to the subtle differences in auxin efficacies. One major difference found in the current study compared to previous work was the lack of binding of quinclorac to any of the receptors (Figs 2 and 3). Analysis revealed that the substance previously tested^18^ did not have the same structure as the confirmed quinclorac sample utilized in the current binding studies.

Foliar applications were utilized to correlate the pharmacological properties of the receptors to biological, whole‐plant responses. *A. thaliana* deficient in AFB2 was not assessed due to the lack of any unique binding affinity relative to TIR1. The three 6‐arylpicolinate analogs induced strong growth reduction on WT *A. thaliana*, whereas the *afb5‐1* mutant showed moderate resistance to all three. Hence, there is a correlation between the binding properties described above and the physiological responsiveness in these cases. However, there is no general correlation between receptor binding *in vitro* and physiological responsiveness *in vivo*. In some cases these discrepancies can be explained, in other cases more information is needed.

The phytotoxicity of 2,4‐D (Table 3) and the root inhibition dose–response data (Fig. 5) both suggest much higher efficacy for this auxin herbicide than would be expected based on the measured binding affinities (KD; Table 2). This mismatch is probably due to the known properties of auxin transporter proteins, which combine to concentrate 2,4‐D inside plant cells, as well as low metabolism, hence multiplying dose–response effects for this compound *in vivo*.^30^ A similar picture emerges from the data for the benzoate, dicamba. In Arabidopsis, the growth reduction of the WT caused by dicamba was much stronger than anticipated based on the very low relative binding to all three of the receptors (Table 3). Not all the parameters of transport are known for dicamba and mathematical models for accumulation are lacking, but we might predict that its efflux is weak, leading to concentration inside cells and consequent high potency *in vivo*.

It is intriguing that picloram and aminopyralid both showed low binding to AFB5 relative to the 6‐arylpicolinates, and yet the *afb5‐1* mutants showed the highest level of resistance to these two compounds based on DW data (Table 3). Again, the phenotype is likely to be a complex function of binding, transport and metabolism given that it has been shown that the auxin uptake carrier protein Auxin1 (AUX1) has a very poor affinity for picloram (it was not determined for aminopyralid)^31^ such that the lack of uptake and absence of its preferred receptor confer high resistance in this case. A further complexity is added by considering the pyrimidine‐carboxylate aminocyclopyrachlor. The DW GR50 of WT *A. thaliana* for aminocyclopyrachlor is similar to that of aminopyralid, and the binding data with all three receptors was similar for picloram, aminopyralid and aminocyclopyrachlor, with highest binding to AtAFB5 (Fig. 3). Yet, the level of resistance of the *afb5‐1* mutants for aminocyclopyrachlor was much lower than for the other two compounds.

The lack of a general correlation between *in vitro* and *in vivo* activities is a key finding and a pragmatic evaluation of all experimental parameters is necessary. The reductionist, biochemical assay is validated by being auxin‐dependent, compound selective and explained in atomic detail by the published protein crystallography results (PDB database entries 2p1m–2p1q and others) and such approaches have proved a satisfactory basis for, for example, structure‐led drug design. Nevertheless, it is not possible to prove that these purified receptor complexes are identical to the native situation. However, while the parameters of the biochemical binding assay are known and controlled, those affecting assays *in vivo* remain largely unknown. For example, different compounds will be exposed to different transport routes and efficacies as well as different metabolic pressures, all possible contributions to the mismatches in activities *in vivo* and *in vitro*.

Auxin‐dependent responses have been found to be triggered by extracellular Auxin‐Binding Protein1 (ABP1)^32^ as well as by the TIR1/AFB receptors. The ABPs signal through trans‐membrane kinases and not *via* TIR1 and this alternative signaling pathway could account for the discrepancies between TIR1/AFB binding and herbicide activity. Unfortunately, there is no receptor binding data for ABP1 and ABP1‐like proteins available for the picolinates. Rapid physiological responses to auxins have also been reported, although the receptor for these is believed to be a member of the TIR1/AFB family, primarily cytoplasmic AFB1.^33^ We have not collected data for AFB1, although changes in the binding pocket from TIR1 and from AFB2 are modest (Table 1) and it is not considered likely that these will change the pharmacology profile greatly. Hence, it is unlikely that the rapid auxin response system accounts for the mismatches between measured binding profiles and herbicidal activity *in vivo*. Nevertheless, it will be necessary to complete far more extensive mapping of response networks in the context of whole‐plant herbicide bioassays before we fully understand differential auxin efficacies.

## 5 CONCLUSIONS

It is clear that the field effectiveness of auxin herbicides is the sum of many contributory factors. The pharmacology of three clades of auxin receptor has been measured in detail and compared, revealing the distinctiveness of the AFB4/5 clade and their high affinity for pyridine and picolinate auxins, especially the 6‐arylpicolinates. The sequences and structures of the receptors have helped explain some of the selectivities of the different clades, and molecular details of the binding pocket have been identified to help inform future rational molecular design. Yet, at field, whole‐plant and atomic levels more information is needed before all features of the differential efficacies of the auxin family can be explained fully.

## CONFLICT OF INTEREST

The work was partially funded by Corteva Agriscience, Indianapolis, USA. The authors declare no other conflict of interest.

## Supporting information


**Appendix S1.** Supporting information.

## Data Availability

The data that support the findings will be available on request to the corresponding author following an embargo from the date of publication to allow for commercialization of research findings.
