# Supplementary material for: The differential binding and biological efficacy of auxin herbicides
Source: Pest Manag Sci. 2022 Dec 16;79(4):1305–15. doi: 10.1002/ps.7294 (PMC10952535; doi:10.1002/ps.7294)
Supplement: Supplementary file 1 — Appendix S1. Supporting information. [file PS-79-1305-s001.docx]

**The pharmacology of binding and differential biological efficacy of auxin herbicides**

**Supplementary Information:**

**Justyna Prusinska^1^, Veselina Uzunova^1^, Paul Schmitzer^2^, Monte Weimer^2^, Jared Bell^2^, Richard Napier^1^**^†^

^1^ School of Life Sciences, University of Warwick, Coventry, CV4 7AL, UK

^2^ Corteva agriscience, Crop Protection Discovery & Development, Indianapolis, IN 46268

^†^ Corresponding author: [**Richard.napier@warwick.ac.uk**](mailto:Richard.napier@warwick.ac.uk)

**Authors contact details:**

[**jusprus@yahoo.com**](mailto:jusprus@yahoo.com)

[**V.Uzunova@warwick.ac.uk**](mailto:V.Uzunova@warwick.ac.uk)

[**paul.schmitzer@corteva.com**](mailto:paul.schmitzer@corteva.com)

[**monte.weimer@corteva.com**](mailto:monte.weimer@corteva.com)

[**Jared.bell@corteva.com**](mailto:Jared.bell@corteva.com)

[**Richard.napier@warwick.ac.uk**](mailto:Richard.napier@warwick.ac.uk)**;**

Supplementary information TABLE 1

Auxin binding pocket sequences with the residue number from AtTIR1 (PDB file 2P1N). The residue selections are based on interpretation of the crystal structure (Tan et al., 2007), giving all the residues in each stretch of backbone from the top of each leucine-rich repeat motif to the base lining the auxin-binding pocket. AFB sequences are based on sequence alignments against AtTIR1 using BLAST (Basic Local Alignment Search Tool at NCBI). Gene-specific substitutions are highlighted in yellow.

|  | 77 - 84 | 344 - 350 | 377-381 | 401 - 406 | 436-441 | 460-465 | 485-490 |
| --- | --- | --- | --- | --- | --- | --- | --- |
| AtTIR1 | PHFADFNL | RVFPSEP | VLYFC | RFRLCI | RLSLSG | MLSVAF | KLEIRD |
| AtAFB1 | PHFADYNL | RVFPSEP | VLYFC | CFRLCV | RLSVSG | MLSIAF | KLEIRD |
| AtAFB2 | PHFADFNL | RVFPSDL | ILYFC | RFRLCI | RLSLSG | MLSIAF | KLEIRD |
| AtAFB3 | PHFADFNL | RVFPSDV | ILYFC | RFRLCI | RLSVSG | MLSIAF | KLEIRD |
| AtAFB4 | PHRADFNL | RIFPFDP | ILYFC | VFRLCI | RLAVSG | TLSVAF | KLEIRD |
| AtAFB5 | PHRADFNL | RIFPFDP | ILYFC | VFRLCI | RLAVSG | TLSVAF | KLEIRD |

**Supplementary Information, Figure 1**


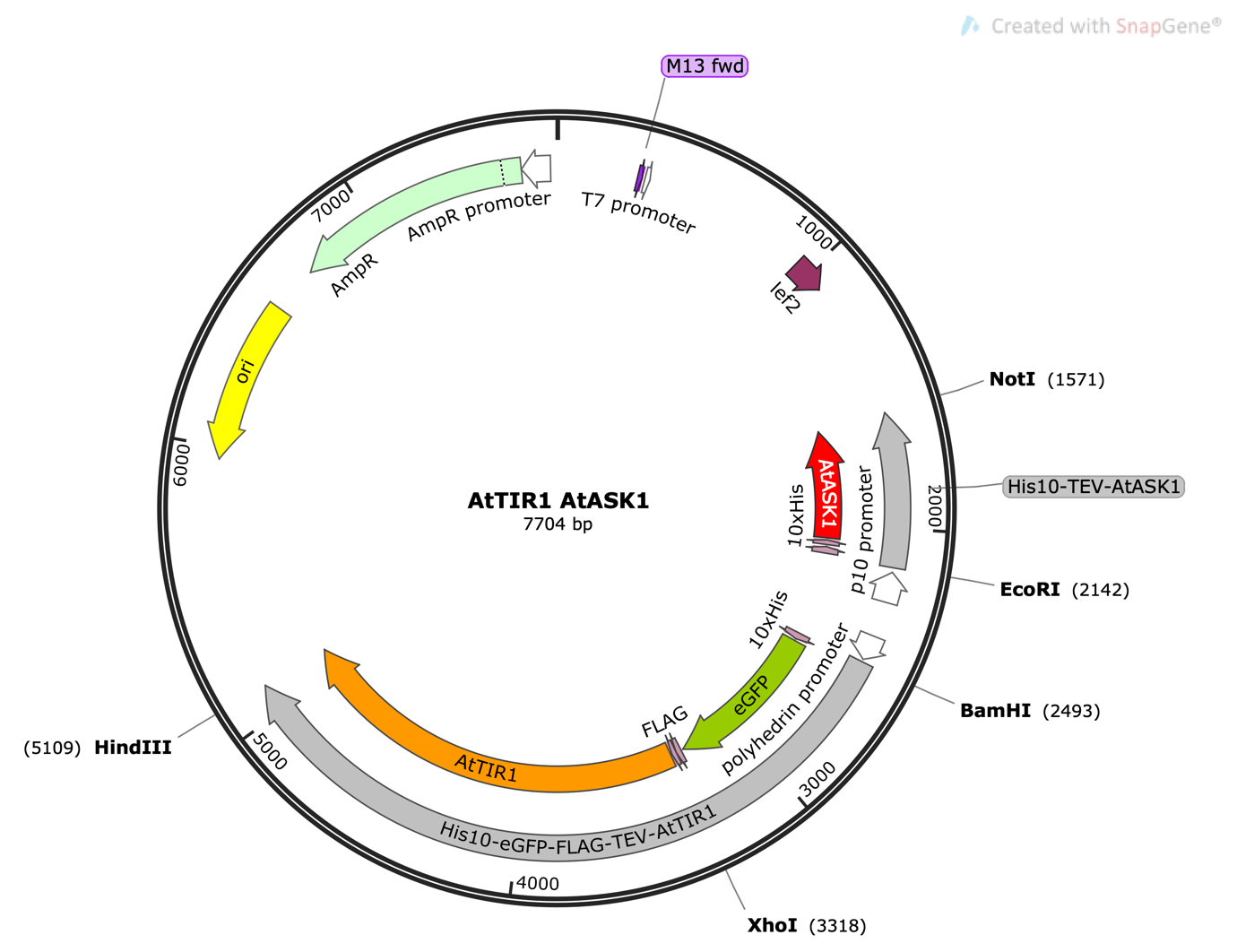


**
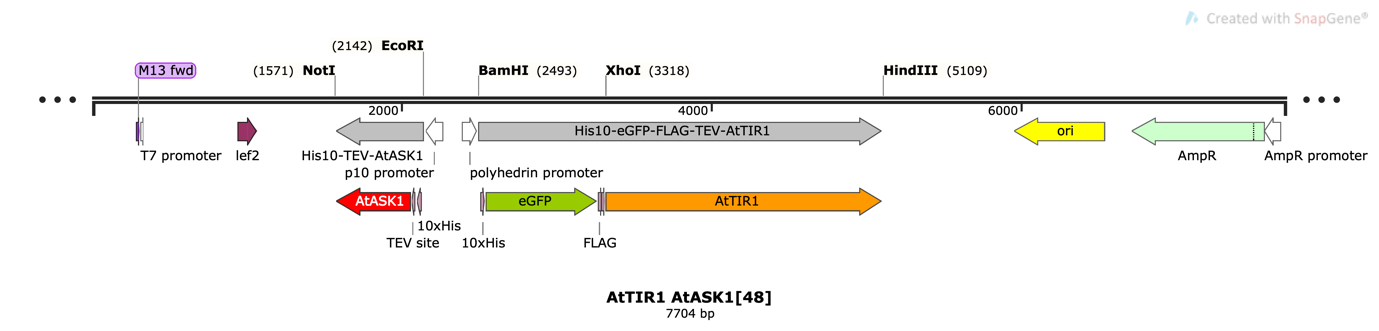
**

**Supplementary information Figure 1.** Example map of baculovirus transfer vector for co-expression of AtTIR1 and ASK. Codon-optimized gBlocks (Integrated DNA Technologies) for His10-eGFP-FLAG-(TEV)-PpTIR1A and His10-(TEV)-PpASK1 were subsequently cloned into the pOET5 transfer vector (Oxford Expression Technologies). The map was created using SnapGene® software (from GSL Biotech; available at [snapgene.com](http://www.snapgene.com/)).

**Supplementary Information, Figure 2.** Purification of receptor proteins.

Proteins were expressed in *Spodoptera frugiperda* (Sf9) cells in tissue culture. An example for AFB5 is shown. Cultures were infected with recombinant baculovirus (Supplementary Information Fig. 1) and cultured for a further 2 days. Cells were harvested by centrifugation, lysed and proteins purified as described in Methods. Samples from representative stages of the purification were separated by SDS-PAGE (equal volumes to indicate yield) and the gel stained for protein with Instant Blue. In the gel image shown, the protein was eluted from FLAG at the end of day 1 and then mixed with the Tobacco Etch Virus (TEV) protease carrying a poly-histidine tag overnight at 5 C (1:1 mole ratio proteins). The mixture was then passed through a new His-Tag column and the unbound proteins collected (lanes 4 and 5). The ASK1 protein has no FLAG tag and so this step removes excess ASK1 protein that is not complexed with TIR1 and the TEV protease to leave receptor proteins affinity tag free.


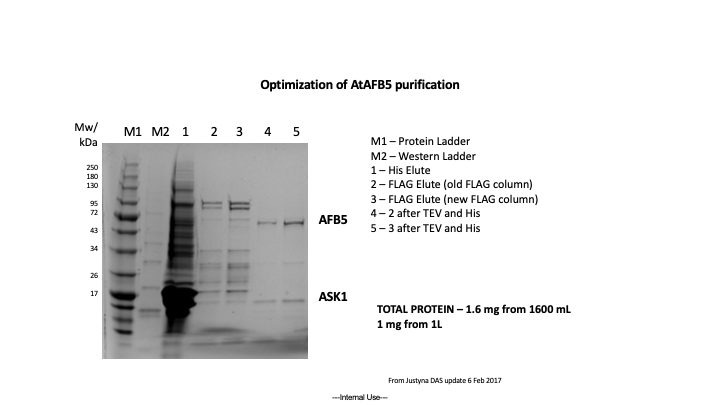


**Supplementary Information, Figure 3.** Comparison of binding to TIR1 and AFB5 for florpyrauxifen, halauxifen, picloram and 2,4-D measured using SPR.


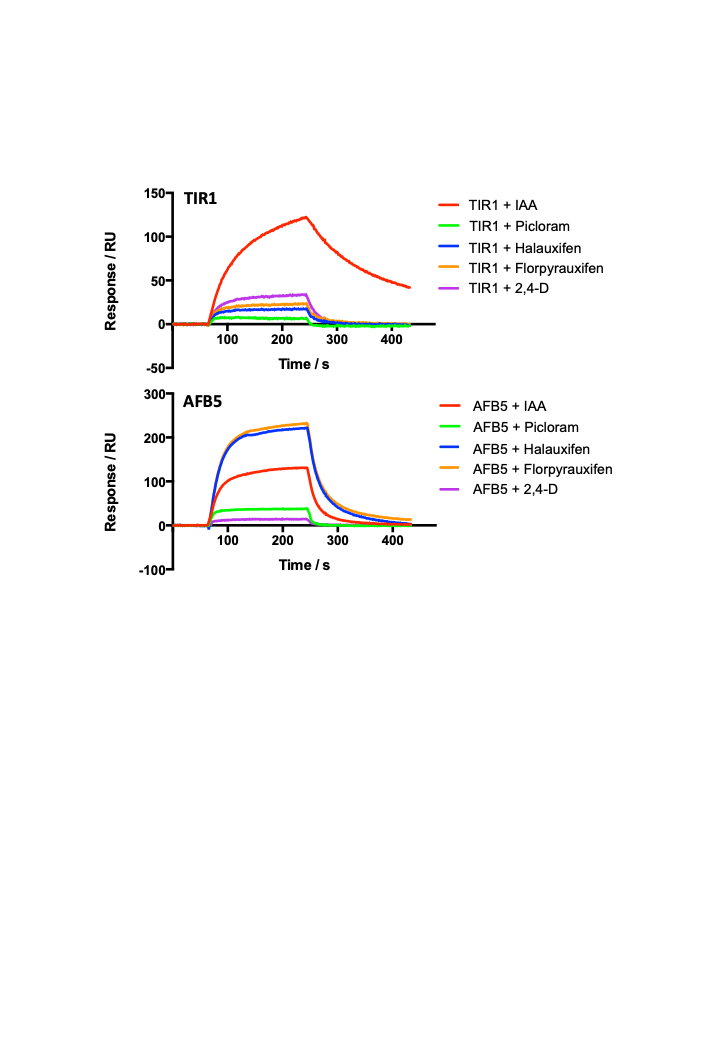


**Supplementary Information, Table 2.** Kinetic data from SPR binding experiments using single cycle kinetics as described in Methods.

**
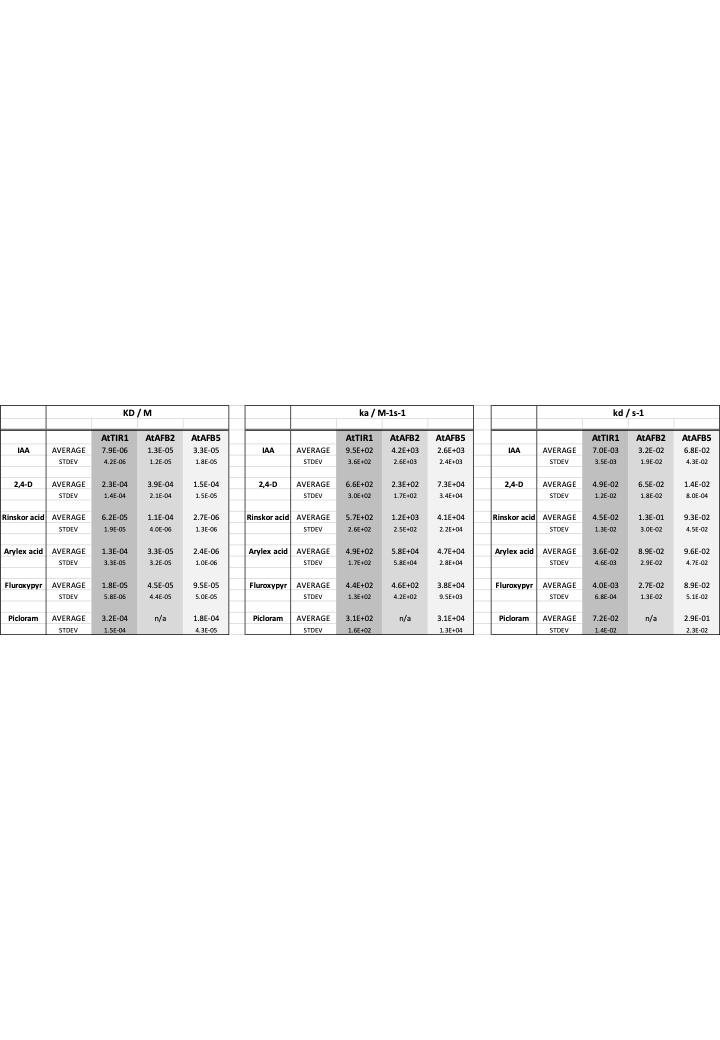
**
